# Exploring the Measurement of Markedness and Its Relationship with Other Linguistic Variables

**DOI:** 10.1371/journal.pone.0157141

**Published:** 2016-06-09

**Authors:** Joanne Ingram, Christopher J. Hand, Greg Maciejewski

**Affiliations:** Department of Psychology, University of Bedfordshire, Luton, United Kingdom; University of Leicester, UNITED KINGDOM

## Abstract

Antonym pair members can be differentiated by each word’s *markedness*–that distinction attributable to the presence or absence of features at morphological or semantic levels. Morphologically marked words incorporate their unmarked counterpart with additional morphs (e.g., “*unlucky”* vs. “*lucky”*); properties used to determine semantically marked words (e.g., “*short”* vs. “*long”*) are less clearly defined. Despite extensive theoretical scrutiny, the lexical properties of markedness have received scant empirical study. The current paper employs an antonym sequencing approach to measure markedness: establishing markedness probabilities for individual words and evaluating their relationship with other lexical properties (e.g., length, frequency, valence). Regression analyses reveal that markedness probability is, as predicted, related to affixation and also strongly related to valence. Our results support the suggestion that antonym sequence is reflected in discourse, and further analysis demonstrates that markedness probabilities, derived from the antonym sequencing task, reflect the ordering of antonyms within natural language. In line with the Pollyanna Hypothesis, we argue that markedness is closely related to valence; language users demonstrate a tendency to present words evaluated positively ahead of those evaluated negatively if given the choice. Future research should consider the relationship of markedness and valence, and the influence of contextual information in determining which member of an antonym pair is marked or unmarked within discourse.

## Introduction

The concept of markedness concerns the relationship between a pair of antonymous or complementary terms. Early formal accounts of markedness would suggest that one member of the pair is physically marked whilst the other is unmarked; semantic markedness would suggest that the words within the pair are differentiated by the presence or absence of a certain property [1, 2, 3]. Morphological markedness, in line with these accounts, occurs when one of the two words is derived from the other through inflection or affixation (e.g., “*lucky*” and “*unlucky*”). Morphologically marked words are defined as unmarked alternatives containing additional morphs. The additional “mark” on one member of a pair of opposites established the notion of markedness [1, 2]. Semantic markedness pertains to antonymous pairs of adjectives, particularly scalar terms [4], in which unmarked members (e.g., “*long*”) are considered to be the default or neutral term in most contexts, whilst the marked member (e.g., “*short”*) cannot be used generically. Within theoretical linguistics literature, the unmarked member, *“long”*, is generally assumed to be more common than the marked member, *“short”* [5]. In the following article we provide a brief overview of theoretical debates on the factors which lead to one of a pair’s members being marked, and consider research which has examined markedness and its effects. We then use an antonym sequencing task to seek to identify the lexical and semantic factors which contribute to markedness.

The examples given above suggest that the marked member of a pair should be simple to identify. However, our consideration of the literature has failed to identify a consensus of simple criteria to define this property. Lyons [6] suggested that if antonyms are morphologically related, morphologically-unmarked terms (e.g., “*pure*” in contrast to “*impure”*) are also considered semantically-unmarked. However, this criterion has several exceptions such as “*independent*” and “*dependent*”. Clark and Clark [7] highlighted three constructions which can be used to identify the unmarked term in a pair of adjectives. First, we are unlikely to ask “*How short is the flight*?” instead of asking “*How long is the flight*?” (n.b., unless the latter question was used deliberately to convey the speaker’s presupposition that the flight will indeed be short). Asking “*How long…*?” does not necessarily pre-suppose the flight to be long, as “*long*” is a default expression. Second, if describing the length of our journey we would state “*The flight was two hours long*” rather than “…*two hours short*”. Finally, when considering the name of a scale, a scale of “length” implies the full scale from the shortest to the longest, while a scale of “shortness” only highlights the negative end of the scale, the end which in comparison to the full size is considered short. Lehrer [8] made similar suggestions, noting that the unmarked member is used neutrally when asking a question, or naming the entire scale, and is additionally more common and associated with the positive meaning of a scale. Horn [3], in a review of markedness and negation, highlighted that within freezes (where the order of two conjuncts presented together within speech is stable [9]; e.g., “*yes”* is generally presented before “*no”*) the unmarked member will be delivered prior to the marked member [10]. Further suggestions were made by Greenberg [11], consistent with the “economy of language” principle [12], that unmarked words should be morphologically and phonologically simpler as they are default [2, 13]. Unmarked items have broad contextual applicability, and appear more frequently than their marked counterparts (e.g., “*possible*” = 336 versus “*impossible*” = 68 occurrences per million words; British National Corpus (BNC), http://www.natcorp.ox.ac.uk). Greenberg [11] suggests that unmarked members of antonymous pairs are the most frequent, simplest, logically-prior, first learned and most natural. Haspelmath [14] argues that all senses of markedness in some way share the concept of markedness as abnormality; however, he also argues that markedness is an unhelpful term which can generally be replaced by more straightforward concepts. Although different, the suggestions made by the authors above do have common themes, most notably those of frequency and valence–valence being whether the word in question is evaluatively positive or negative in correspondence to the semantic factor of evaluation [15]. We may conclude from these discussions that a marked word will be both less frequent than its unmarked counterpart and more negative in valence. As these prior discussions have been for the most part theoretical, the question posed here is whether or not markedness in itself is an independent property which influences language processing. When a word within a pair is labeled as “marked”, is this simply a reference to the less common word, the more negative word, or is there an additional property which the current label of “marked” represents?

We have been unable to identify prior research detailing a direct, experimental assessment of markedness. That is, we do not know of any studies which attempt to establish word markedness as it may be represented internally by speakers / readers. However, there are studies which have assessed the effects of markedness on other processes. Reasoning problems posed using the unmarked member of a word pair were solved more quickly and with fewer errors than those using the marked member, suggesting that problems involving unmarked terms require less memory load during processing [16, 17]. According to Clark [16], these results suggested that valence was a key feature in the determination of markedeness. Notably, words with a positive meaning (e.g., “*good*”) were held in memory in a less complex way than their negative antonyms (e.g., “*bad*”). As the positive term is also the default, it is easier to retrieve from memory than the negative term which is only available as a contrastive. That is, “*bad*” may only be stored to represent a contrast to “*good*” rather than as a default situation. This possibility is supported by a more recent study of discourse processing where Fraenkel and Schul [18] found that meaning mitigation–negated adjectives (e.g., “*not hot*”) conveying weaker meaning than their corresponding antonym (e.g., “*cold*”)–is more pronounced when such negated adjectives are marked terms. Furthermore, when comparing marked and unmarked adjectives, the extent of meaning mitigation is greater in negated marked adjectives than their unmarked equivalents (e.g., “*not cold”* vs. “*not hot”*). These results underline the possibility that markedness influences on-line written language processing. In order to operationalize markedness, these authors used the criteria laid out by Lehrer [8] while also noting that in previous studies negativity has been identified as an indicator of markedness [7, 8, 19]. One question which then arises is whether or not markedness is merely another manifestation of valence. As noted there are examples of negative words which are unmarked (e.g., “*selfish*”; see S1 Dataset), therefore one aim of the current paper was to more clearly establish to what extent markedness and valence are separate properties.

The notion that words evaluated negatively are used differently in language to those evaluated positively is not particularly novel. The Pollyanna Hypothesis [19] suggests that positively evaluated words will differ from negatively evaluated words in many of the same ways as unmarked words are considered to differ from marked words. In a cross-cultural investigation using a word association task, these researchers found that positively evaluated words were generally found to be used more frequently and diversely, and were more likely to have a negative affix applied than negatively evaluated words. These results were supported by developmental data [20], as positively evaluated words were found to appear earlier in language development. A linguistic explanation for the preference of positively over negatively evaluated terms has been proposed in terms of the “Pollyanna principle” [21]. However, there is little, if any, further empirical work that discusses the validity of the Pollyanna Hypothesis.

A preference for positively evaluated terms was found in a correlational study by Zajonc [22]; however, this research highlighted the effects of mere exposure as opposed to polarity of evaluation. Zajonc [22] asked participants to assess 154 antonym pairs and identify which word within each pair had a more favorable meaning or word referent. For 82% of pairs, the more desirable word within the pair was also the more frequent. Interestingly, Zajonc’s [22] data includes a percentage of agreement which allows us to see how many of the 100 participants selected the word as more favorable. There is some evidence that for pairs where agreement between participants is lower with regards to the more favorable word (i.e., where participant agreement is closer to 50%), there was less variation in word frequency between the two words in the pairs. The studies which established the Pollyanna Hypothesis and the Mere Exposure Effect allow us to consider two things. First, although the above researchers agree that they are essentially demonstrating the same relationship between valence and frequency, there is no consensus with regards to causality. The Pollyanna Hypothesis suggests that the words are more frequent owing to their positivity, whereas the Mere Exposure effect suggests that by being frequently encountered, a word becomes more positively appraised. Boucher and Osgood [19] provide criticism of Mere Exposure by noting that repeated exposure to unfamiliar materials may lead to preference. However, the same cannot be said for familiar words as, if this were the case, then high frequency words such as “*pain*” would eventually develop a positive interpretation. Our second consideration is that if frequency and valence are the key predictors of the markedness of a word, is the property of markedness simply a binary classification of the effects demonstrated by Boucher and Osgood [19] and Zajonc [22]?

We sought to assess markedness independently of the other factors detailed above (e.g., desirability), before assessing if this measurement of markedness was related to other word factors. It was previously noted that where the order of two conjuncts in speech is stable the unmarked member will be presented prior to the marked member [8]. Recent corpus research on the sequence of antonyms [23] suggested that the order in which antonym pairs are presented when they jointly occur in discourse is strongly correlated with the concept of markedness. Kostic [23] examined 57 antonymous word pairs within representative contexts in written Serbian discourse. Forty-three of the pairs exhibited a statistically significant preference to a specific word ordering (e.g., A followed by B or B followed by A). This finding was in contrast to previous research [24] where markedness was not considered as a cause of antonym sequences. Jones [24] implied that markedness is represented only by semantic neutrality. As this factor overlapped with positivity (valence) and morphology–with respect to their influence on antonym sequences–it was determined to be inconsequential. However, Kostic [23] notes that the factors Jones [24] identified as overlapping were the same factors which, in a less simplified description, are considered to determine markedness. The more recent corpus study [23] suggests that the criteria that determine antonym sequences in discourse (word frequency, valence, morphology, neutrality and quantity) are the same as those that can be used to determine markedness. As a result, it could be said that markedness determines antonym sequences. In order to assess markedness at the level of words rather than discourse we developed a paradigm similar to that of Zajonc [22], which focused on the sequence of antonym pairs rather than explicit preference.

Participants were asked to recombine antonym pairs which were randomly split across two sheets of paper. After a pair had been selected, rather than identifying the more desirable or preferred member of the pair, participants were asked merely to report pairs. The probability that a word is reported first in the pair acted as an indication of markedness. Instructions intentionally avoided giving participants any indication of how they should select the order of the words, so as to minimise any direct consideration of the valence, frequency, or morphology of words when ordering. This paradigm generated results that indicate whether markedness can be considered a dichotomous variable, or if words vary along a dimension of markedness. If markedness probabilities are for the most part dichotomous, this would suggest that use of the term “marked” is a binary classification of words that hold certain properties, including negative valence and low frequency. If markedness probabilities are more variable, this may suggest that markedness is an additional property which may be related to–but is not reliant on–other factors.

Lyons [5] highlighted that words vary in their degree of semantic markedness; within the pair “*dog*” and “*bitch*”, the female member is highly marked as the male member is so commonly used as the default. The degree of markedness is less pronounced for the male in the pair “*cow*” and “*bull*” despite the female counterpart being the default. Alternatively, Cruse [25] does not agree that there are degrees of markedness as a marked member may only be used as an implicit or explicit contrast to the unmarked member; that is, “*bad*” is used to signify “*not good*” rather than indicating an entirely separate state. Further analysis of the data collected using the current paradigm indicated the extent to which a word’s degree of markedness can be predicted by a number of other variables, most notably those argued to reflect markedness [11] and those thought to determine antonym sequences in discourse [23]. Theory suggests that those words which are strongly negative and uncommon (i.e., low frequency) are generally marked, so we hypothesised that words with a low valence rating and words which are less frequent will be highly marked, as indexed by markedness probability derived from the antonym sequencing task.

## Method

### Design and Materials

Markedness probability was measured using an antonym sequencing task (see *Procedure* for further details). The materials consisted of 154 word pairs taken from a previous study by Zajonc [22]; word pairs are detailed in S1 Dataset. Pairs were divided randomly across two A4 sheets, and a Microsoft Excel spreadsheet was used to collect participants’ responses. Markedness probability was then used as the dependent variable in a general linear model with the independent variables: raw and log-transformed word frequencies [26], word valence ratings for each word (provided by 50 additional independent participants; -3 = completely negative, 3 = completely positive), word length (letter count), morphological status (affixation), number of orthographic neighbors [27], age of acquisition [28] number of word senses [29], and word preference ratings [22].

### Participants

Fifty native English-speakers took part in establishing markedness probabilities. Before taking part in this research, all participants provided informed written consent by signing a consent form which highlighted that participation would be confidential and anonymous. The consent procedure, consent form, and research protocol was approved by the Department of Psychology Ethics Committee at the University of Bedfordshire.

### Procedure

Participants were presented with 154 antonym word pairs, taken from Zajonc [22]. Antonym pairs were pseudo-randomly allocated across two A4 sheets, with one pair member appearing on Sheet A and its partner on Sheet B. The location and order of words on these sheets remained the same for all participants. The two sheets were presented to participants side by side with half of participants receiving Sheet A to their left and half of participants receiving Sheet B to their left. Participants matched words from each sheet into their antonym pairs. Participants were instructed to carefully consider the order in which they reported each member of the pair; they were asked to enter the words into columns A and B of a two-column Microsoft Excel spreadsheet in what they felt was the most appropriate order. Participants were given no further information on how they should decide on the order of the words. Unlike previous measures of markedness [22], instructions were written to avoid mentioning word preference or commonality, as judgments made with these factors in mind might reflect valence and frequency as opposed to markedness. Misspelled words were excluded from analyses (3.39% of the data obtained). The likelihood of a word being entered first in the pair constituted markedness probability. High-probability words (0.51–1) were interpreted as *unmarked*, and low-probability words (0–0.5) interpreted as *marked*. Our justification is that in Column A, participants provided us with the words they would normally present first or consider the default, as opposed to those that they necessarily prefer or find more desirable.

## Results

All items were split into semantically- (*n* = 172, e.g., “*near*” vs. “*far*”) and morphologically-determined markedness categories (*n* = 136, e.g., “*fasten*” vs. “*unfasten*”) based on the theoretical forces underlying word markedness (see Introduction). Markedness data showed that although there were some items that had a markedness probability of approximately 0.5, most of the words had either a notably high probability or a notably low probability (for the stimulus list, information on additional lexical and semantic variables, and classifications of the stimuli, please see S1 Dataset). Strongly marked (probability between 0 and 0.25) and strongly unmarked (0.75 and 1) words cumulatively constituted 73% of the semantically determined items and 93% of the morphologically determined items. This pattern of data demonstrates that most of our stimuli could be classed as either strongly marked or unmarked. We compared the obtained markedness probability to theoretical markedness of the stimulus set–a binary classification of markedness where words with probabilities from 0–0.5 are classed as marked and those with probabilities of 0.51–1 classed as unmarked. A logistic regression showed that, irrespective of markedness type, the obtained markedness probability (*B* = 7.48, *SE* = 0.96) could correctly class 93.5% of the items as either marked or unmarked antonyms [χ^2^(3) = 321.05, *p* < .001; Cox & Snell *R*^2^ = .65, Nagelkerke *R*^2^ = .86].

Although it is logical that unmarked words are also the more preferred, our measure of markedness probability is substantially different from explicit word preference ratings. We conducted a multiple regression with both markedness probability, markedness type (semantic vs. morphological), and their interaction as predictors of Zajonc’s [22] word preference ratings. The model showed that markedness probability (*B* = 89.35, *SE* = 5.30, *t* = 16.87, *p* < .001), independently of markedness type (*B* = -0.73, *SE* = 4.48, *t* = -0.16, *p* = .87), accounts for only 67% of variance in word preference ratings [*F*(3,304) = 209.84, *p* < .001, *R*^2^ = .67]. This suggests that our markedness probabilities are not another measure of word preference. In order to investigate what word properties underlie the present measure of word markedness, as opposed to word preference, markedness probability was analysed in conjunction with a variety of established lexical (word frequency, length, affixation, and number of orthographic neighbors) and semantic variables (number of word senses, age of acquisition, and valence). Bivariate correlations showed that markedness probability was associated with a number of these variables. For semantically determined items (*n* = 172), markedness probability correlated with log-transformed word frequency (*r*_*s*_ = .19, *p* < .01) and valence (*r*_*s*_ = .65, *p* < .001). For morphologically determined items (*n* = 136), markedness probability correlated with log-transformed word frequency (*r*_*s*_ = .59, *p* < .001), length (*r*_*s*_ = -.48, *p* < .001), affixation (*r*_*s*_ = -.87, *p* < .001), number of orthographic neighbours (*r*_*s*_ = .30, *p* < .001), number of word senses (*r*_*s*_ = .39, *p* < .001), age of acquisition (*r*_*s*_ = -.19, *p* = .01), and valence (*r*_*s*_ = .79, *p* < .001). All other variables did not significantly correlate with markedness probability. These initial results guided subsequent analyses.

To delineate the characteristics of marked and unmarked words, we conducted a multiple regression for semantically- and morphologically determined markedness separately. All predictors were centered (the mean of a variable was subtracted from each observation’s raw value) to minimise multi-collinearity and to make regression coefficients more interpretable. The predictors were entered in steps. The initial step consisted of lexical-level variables: log-transformed word frequency, length, number of orthographic neighbours, and affixation for morphologically determined items only. Further variance in markedness probability was then accounted for by predictors associated with semantic features: number of word senses, age of acquisition, and valence. For semantically determined items (see Table 1), word valence was the only significant predictor of markedness probability [*F*(6,165) = 20.74, *p* < .001]. When all other variables are held constant at their means, each one-unit increase in valence predicted markedness probability to rise by .19, demonstrating that unmarked words (e.g., “*near*”) tend to be more positive than the marked member of their antonym pair (e.g., “*far*”).

**Table 1:** Linear regression model of markedness probabilities for semantically determined items.

*Predictor variable*	*β*	*p*
Step 1: Lexical variables		
Length	<-.01	.98
Log-transf. frequency	.04	.26
Neighborhood size	< .01	.38
*Adjusted R*^*2*^	.*05*	
Step 2: Semantic variables		
Word senses	<-.01	.62
Age of acquisition	.01	.25
Valence	.19	< .001
*Adjusted R*^*2*^	.*41*	

For morphologically determined items (see Table 2), markedness probability was best explained by both affixation and valence [*F*(7,128) = 392.56, *p* < .001]. As in the previous model, the coefficient associated with valence suggests that, regardless of other word properties, words with more positive meanings (e.g., “*pleasant*”) are more likely to be unmarked than their more negative counterparts (e.g., “*unpleasant*”). The model also shows that when valence is held constant, having a prefix predicted markedness probability to *decrease* (become more marked) by .65. Although this effect is inherently related to word length (*p* = .60) and the frequency of the orthographic word form (*p* = .19), the model suggests that word stems (e.g., “*fasten*”) are more likely to be unmarked than words with a prefix (e.g., “*unfasten*”).

**Table 2:** Linear regression model of markedness probabilities for morphologically determined items.

*Predictor variable*	*β*	*p*
Step 1: Lexical variables		
Affixation	-.65	< .001
Length	<-.01	.60
Log-transf. frequency	.02	.19
Neighborhood size	.01	.40
*Adjusted R*^*2*^	.*94*	
Step 2: Semantic variables		
Word senses	< .01	.31
Age of acquisition	<-.01	.17
Valence	.05	< .001
*Adjusted R*^*2*^	.*95*	

These results demonstrate a relationship between markedness and affixation and a relationship between markedness and valence. The relationships between both lexical and semantic variables and markedness probabilities reflect the postulates of the aforementioned linguistic theories of markedness. Our results suggest that markedness is not a strictly binary concept. We note that the majority of the words within our study can be classified as having a distinctly high or low markedness probability; however, there is still considerable variation within the markedness probabilities obtained that deviate from a strictly binary perspective. The probability that a word is marked / unmarked is variable and this variation is clearly related to the valence of a word, particularly when there is no morphological mark. What is less clear is whether markedness probability, measured using an antonym sequencing task, is merely a manifestation of affixation and valence, or if this measure suggests that markedness probability may be related to but not determined by these variables.

## Discussion

The present paper uses an antonym sequencing task to quantify the probability of markedness for each word within a pair of antonyms. We then examined the relationship between this markedness probability measure and other linguistic factors, determining which factors may be used to predict the markedness probability of a word. Analyses suggest that the property of markedness may be strongly related to the valence of a word and to whether or not the word contains an affix.

Early accounts of markedness referred only to those word pairs in which members were morphologically differentiated [1], and it is clear from the current results that a word being affixed makes it more likely to be the marked member. Words without affixes can also be considered simpler, and hence these results support, in part, the suggestions of Greenberg [11]. Grammaticalization supposes that a process of phonetic reduction, as a response to frequency of use [30], leads to more frequent words being morphologically reduced. This possibility is supported by the correlation between markedness probability and word frequency for morpholocially-determined items. However, whether a given word was highly marked or not did not reliably depend on its word-form frequency, contrary to the intuitive assumptions made in the theoretical linguistics literature [4, 11]. There is a trend suggestive of a relationship between the two word properties; however, frequency failed to account for markedness probability over and above valence.

We hypothesised that there would be a significant relationship between valence and markedness based on theoretical discussions of the concept. Although linguistic descriptions of markedness have referred to a range of ways to identify the marked member, the property of negativity had been considered a strong enough indicator of markedness to partially drive material selection in other studies [18]. Previous research by Zajonc [22] closely linked valence to word preference. Zajonc [22] asked participants to identify the more desirable member of pairs of antonyms. Words identified as unmarked in the current study (high markedness probability) reflect the desirable words of Zajonc [22]–as shown by a correlation between our data and Zajonc’s preferences. Previous discussion also led us to hypothesise that there would be a significant relationship between frequency and markedness. Greenberg [11] suggested that unmarked terms appear more frequently, and Zajonc [22] noted that the desirability rating of antonym pairs was likely to be strongly linked to exposure. That is, the more we are exposed to a word the more desirable it becomes. In the present study, there was a simple correlation between markedness and frequency, but frequency was not found to be a significant predictor of markedness probability. This suggests that although unmarked words are more frequent, they are not necessarily unmarked *because* they are more frequent. As our markedness probabilities do not perfectly mirror the “desirability” ratings determined within Zajonc’s [22] study, and as frequency is not a significant predictor of markedness probability, our results lend support to the Pollyanna Hypothesis, based on the effect of valence on markedness probability observed in the current paper. In their research on the Pollyanna Hypothesis, which suggests that evaluatively positive words are used more frequently and diversely in language than evaluatively negative words, Boucher and Osgood [19] linked many of the factors theoretically assumed to account for the relationship between markedness and word valence. That is, those words which are evaluated more positively are more likely to be unmarked–theoretically, more likely to be more frequent, of simpler construction, and used as the default.

We argue that although interrelated, our markedness probability score is not merely another measure of valence. Marked words are those 154 words with a markedness probability of <0.5 –however of these words only 77% have a negative valence rating. Valence may be an important factor in markedness, but marked words are not exclusively negative, and, to a lesser extent, unmarked words are not necessarily positive (12% of words with a markedness probability of >0.5 have a negative valence rating). There are also examples where the linguistic distinction between the marked and unmarked terms is clear, but this is not supported by the markedness probability. Consider the antonym pair “*full”* and “*empty”*. In linguistic terms “*full*” would be considered the unmarked term–it is used generically [4] and covers the entire length of the scale [6]. Valence ratings suggest that “*full”* is somewhat positive (0.98), whilst “*empty”* is somewhat negative (-1.36). “*Full”* also occurs far more frequently than “*empty”* (322.35 vs. 53.65 uses per million words). Yet, the markedness probabilities of these two words are not as widely different as may be anticipated (0.62 vs. 0.38). Such examples (e.g., “*independent*” vs. “*dependent*”, see S1 Dataset) support our argument that markedness is not simply another measure of valence. We argue that exceptional antonym pairs, such as “*lost”* vs. “*found”*, and “*full”* vs. “*empty”* detailed above, may account for our model for semantically determined items explaining only 41% of the variance in the markedness data.

Our results also support recent research [23], which suggests that the ordering of antonym pairs within discourse is related to markedness. By asking participants to determine the order of the word pairs we were able to establish a measure of markedness that is independent of context. Analysis of the markedness probabilities determined that those features which overlapped with markedness in previous research [24] did play a significant role in determining markedness probabilities. We suggest that rather than considering markedness inconsequential in antonym sequences due to this overlap, these features interplayto determine markedness, which determines antonym sequences in turn. To further support this point, we examined the order in which the members of our 154 target word pairs occurred within the British National Corpus [31] (see S2 Dataset for the additional data). We examined sentences in which both the unmarked and marked words (as determined by our measure of markedness probability) were used in order to see which of the terms occurred first most frequently. The search criteria were such that the two antonymous words must occur within a single sentence and within 18 words of one another. Of the 154 possible pairs, 147 were found to co-occur within these parameters (see S2 Dataset). Interestingly, all seven that were not included were morphologically-marked pairs. Our search returned 122,519 instances of collocation across the target word pairs. According to our classifications of markedness probability, unmarked members of the target word pairs occurred first in 65.1% of these 122,519 cases. In order to explore this at the level of each member of the 147 pairs, we considered for how many of these pairs the unmarked term occurred before its marked partner. We found that in natural language the unmarked member of the pair was more likely to appear first for 122 of the 147 pairs examined (83.0%) whereas the marked member was more likely to be first in only 21 cases (14.3%) with 4 ties (2.7%).

In order to further explore our measure of markedness within natural occurrences of collocated antonyms, we considered the dominance of unmarked terms and separated the 147 collocated pairs into those which were semantically determined and those that were morphologically determined. Within the corpus, semantically unmarked antonyms were more likely to occur first in a collocational part of discourse compared to their marked counterparts. As determined by our markedness probabilities, semantically unmarked antonyms dominated (i.e., were more likely to occur first) in 87.4% of pairs when collocated with their marked partner; whereas the marked term (e.g., *“late”* followed by *“early”*, *“outside”* followed by *“inside”*) predominantly occurred first in only 11.5% of the collocational pairs (1.2% ties). A chi-square analysis of these data revealed that the distribution of observed frequencies was significantly different from chance, suggesting that there is a relationship between markedness and the likelihood that a member of a semantically determined antonym pair will occur first in natural language [χ^2^ = 183.21, *df* = 3, *p* < .001].

When considering morphologically determined antonyms, this dominance of unmarked terms was smaller, suggesting that the positions of morphologically marked and unmarked antonyms are more interchangeable than those of semantically determined antonyms. Morphologically unmarked antonyms occurred predominantly first in 68.7% of the pairs, whereas the morphologically marked terms (e.g., “*inadequate”* followed by “*adequate”*, “*unlikely”* followed by “*likely”*) occurred first in 16.4%, with 4.5% ties, withthe remaining pairs not occurring collocationally (10.5%). Although a chi-square analysis of these data revealed that the distribution of observed frequencies was significantly different from chance, suggesting that there is a relationship between markedness and the likelihood that a member of a morphologically determined antonym pair will occur first in natural language [χ^2^ = 70.01, *df* = 3, *p* < .001], this relationship appeared to be weaker than that for semantically determined antonym pairs and their use in natural language. Greater interchangeability in the position of pair members for morphologically marked pairs reflects that a common ‘stem’ is present in both members of the pair–therefore the reader / listener encounters the core concept regardless of the order in which pair members are presented (e.g., valid / invalid–*validity*). If members of antonym pairs are differentiated semantically, without a more abstract core concept, word order may serve an important function, facilitating the processing of the developing discourse (e.g., husband / wife–*family relationship*). Based on a qualitative analysis of morphologically and semantically determined word pairs, we identified no systematic pattern that would explain why both morphologically and semantically marked terms would sometimes occur before their unmarked counterparts. In a few instances, a word’s markedness in isolation seems to slightly differ from the one in discourse, possibly reflecting the highly variable nature of contexts in which one uses two antonyms within the same sentence.

We then proceeded to examine the markedness measure of all 294 words that occurred collocationally with their individual likelihoods of occurring first in discourse, with the prediction of a strong positive correlation between our markedness measure and likelihood of occurring first within discourse. A one-tailed Pearson’s correlation provided clear support for the relationship between our measure of markedness and likelihood of first occurrence in collocational discourse (*r* = .6, *N* = 294, *p* < .001). We further examined in isolation the relationship between markedness and first-occurrence likelihood for semantically and morphologically determined antonym pairs. Correlations were significant for both semantically- and morphologically-determined items (semantic: *r* = .73, *N* = 172, *p* < .001, morphological: *r* = .62, *p* < .001), thus demonstrating that our measure of markedness probability is reflected in natural written discourse (or that prior experience with natural written discourse informed our participants’ assessments of word pair ordering). These results lend support to the suggestion that the unmarked member will precede the marked member in freezes [3, 9, 10]. However, as with the number of cases where the unmarked term showed dominance in the likelihood of first occurrence, the bias for semantically determined terms was stronger than for morphologically marked terms, suggesting more flexibility in the word order for such morphologically determined antonym pairs.

In the present experiment, we quantified the markedness of antonymous words and examined its relationship with other psycholinguistic variables using an antonym sequencing task. We developed and used this novel paradigm for two reasons. First, it was crucial to examine native speakers’ tendency to use one antonym before another without explicitly asking them to select the preferred member of the antonym pair. The antonym sequencing task minimises the natural confound between word markedness and preference, as demonstrated in the analysis contrasting markedness probabilities and Zajonc’s [22] preference ratings. Second, the task has also allowed us to establish, for the first time, the markedness of words presented in isolation, i.e., words encountered outside of discourse. Since context plays a substantial role in determining whether a given antonym is marked or unmarked [32], it was crucial to develop a task that would reduce this bias. The data we provide represent the scores of “default” markedness that possibly generalise across a variety of contexts in which the antonyms can appear. This is evident in the analysis of the antonym sequences in discourse. While the markedness probability data could reliably predict which of the antonyms precedes its counterpart in most of the extracts, there were a few instances, particularly for morphologically determined markedness, in which it was the marked antonym that was used first. Overall, the antonym sequencing task has produced reliable estimates of the markedness of antonyms that are free of contextual bias and participants’ preferences for certain words. We developed this paradigm to specifically address our research aims. As there are no alternative experimental measures of markedness we are unable to provide a comparison of our measure with those of others; however, there is considerable evidence to suggest that the task does capture the nature of markedness. Our markedness probabilities mirror the linguistic classification of the antonyms into marked and unmarked ones based on the theoretical principles underlying markedness (e.g., valence, affixation), and reflect the ordering of antonyms in natural language, as demonstrated in the analysis of the corpus data. The sequencing task provides a suitable empirical measure of the theoretical concept of markedness; it reflects participants’ genuine tendency to use one word before another.

With regard to future studies, our results suggest that when dealing with word valence, researchers should proceed with caution to ensure that valence is actually the property under investigation. In particular, we suggest ensuring the counterbalancing of markedness and valence. For each word pair where markedness and valence correspond (e.g., *“hot”* vs. *“cold”*), an additional pair where these factors do not correspond (e.g., *“lost”* vs. “*found”*) should also be examined, to ensure that effects attributed to valence are not actually brought about by markedness.

To summarise, our results suggest that markedness is independent from word frequency, but closely related to valence. These results provide support for the Pollyanna Hypothesis and for the suggestion that markedness guides the ordering of antonym pairs within discourse. The current paper focuses on marked and unmarked words in the absence of context; however, in language processing contextual information establishes what is expected in a given discourse, and may also determine which member of a pair is the unmarked term. For example, the concept of “warmth” may be unmarked in the context of a summer vacation, but it will become marked in the context of a ski trip [32]. Further research may wish to address the effect of contextual predictability on the processing of marked and unmarked words using eye-movement recording techniques. Studies of eye-movements provide spatially and temporally fine-grained measures of online processing [33, 34]. Characteristics of words, such as valence, frequency, and predictability from context have been found to influence processing load during natural reading [35, 36]. Processing of words which are matched with respect to valence, word-form frequency, and markedness may be evaluated within highly predictable and neutral scenarios in order to establish how contextual information interplays with these different properties of words.

## Supporting Information

S1 Dataset One hundred and fifty-four antonym pairs with markedness probabilities, word classifications and lexical and semantic variables used in the data analysis. (XLSX)

S2 Dataset Collocation data for one hundred and fifty-four antonym pairs with markedness probabilities and types. (XLSX)
